# Dynamic Skin Patterns in Cephalopods

**DOI:** 10.3389/fphys.2017.00393

**Published:** 2017-06-19

**Authors:** Martin J. How, Mark D. Norman, Julian Finn, Wen-Sung Chung, N. Justin Marshall

**Affiliations:** ^1^Ecology of Vision Group, School of Biological Sciences, University of BristolBristol, United Kingdom; ^2^Marine Sciences, Museum VictoriaMelbourne, VIC, Australia; ^3^Sensory Neurobiology Group, Queensland Brain Institute, University of QueenslandBrisbane, QLD, Australia

**Keywords:** dynamic patterns, cephalopod, communication, camouflage, motion, chromatophore, skin, passing wave

## Abstract

Cephalopods are unrivaled in the natural world in their ability to alter their visual appearance. These mollusks have evolved a complex system of dermal units under neural, hormonal, and muscular control to produce an astonishing variety of body patterns. With parallels to the pixels on a television screen, cephalopod chromatophores can be coordinated to produce dramatic, dynamic, and rhythmic displays, defined collectively here as “dynamic patterns.” This study examines the nature, context, and potential functions of dynamic patterns across diverse cephalopod taxa. Examples are presented for 21 species, including 11 previously unreported in the scientific literature. These range from simple flashing or flickering patterns, to highly complex passing wave patterns involving multiple skin fields.

## Introduction

**Figure 1:**
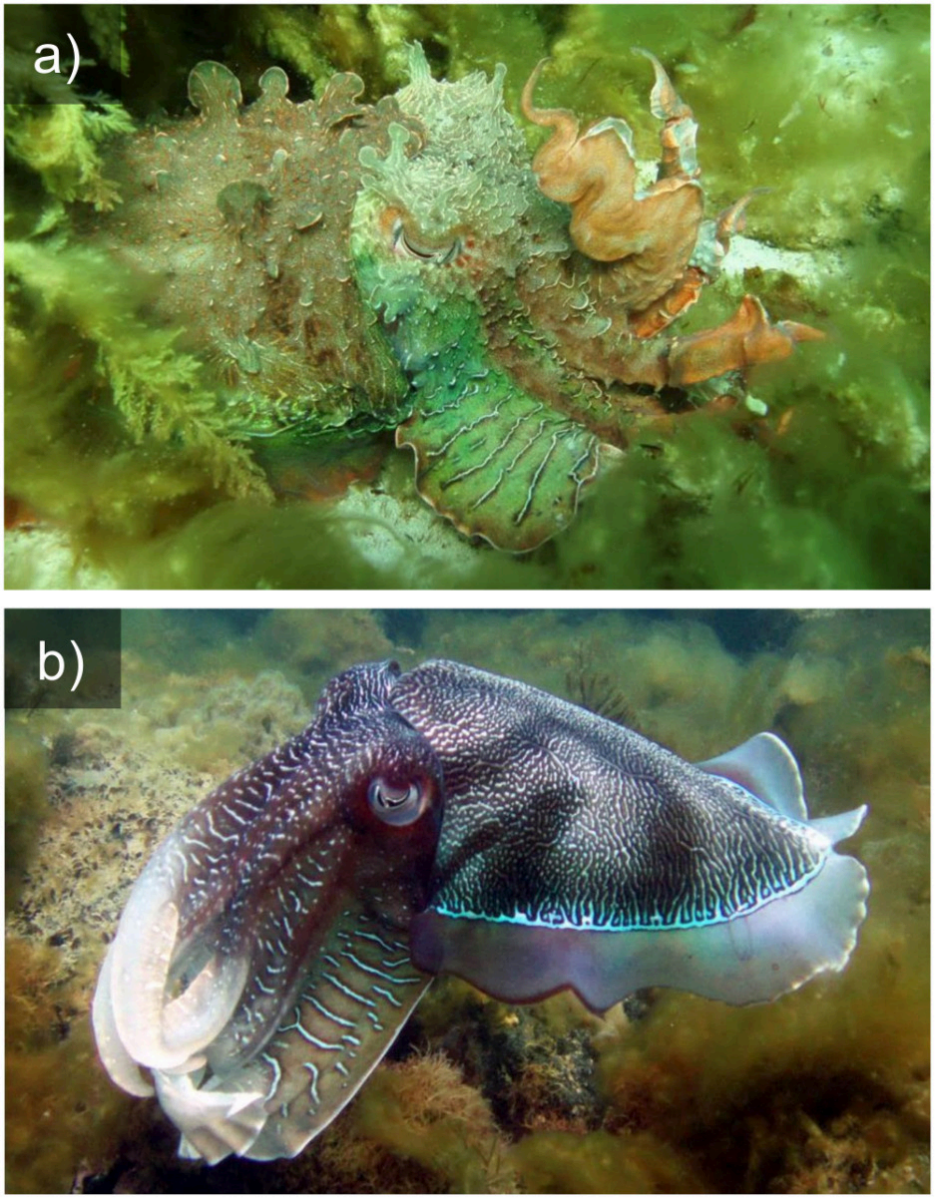
Camouflage **(a)** and signaling **(b)** in the Giant cuttlefish *S. apama*.

Cephalopods are well-known masters of camouflage, but are also unsurpassed in their ability to alter their visual appearance for communication. The most complex of the Mollusca, they have evolved a sophisticated system of neurally- and hormonally-driven active dermal units that produce variable body patterns using three distinct visual components: (1) a chromatic component provided by elastic pigment-filled structures, the *chromatophores*, (2) a color-reflective component effected by wavelength interference platelet structures, the *iridophores*, and (3) a passive reflection component produced by the *leucophores* (Messenger, 2001). Skin patterns in many cephalopods are further enhanced by a textural component, where muscular and hydrostatic forces within the architecture of the skin enable simple to complex changes in skin topography. Amongst benthic octopuses (family Octopodidae) and cuttlefishes (family Sepiidae), this variable sculpture can include flaps, ridges, and/or simple to multiple branching papillae (e.g., Figure 1a).

This unique dermal architecture enables many cephalopods to switch easily between matching the tone and texture of various backgrounds (Figure 1a), through to performing conspicuous signaling displays for intra- and inter-specific communication (Figure 1b). This is particularly impressive considering that the vast majority of cephalopod species are color blind, possessing only a single visual pigment and failing to demonstrate color vision in behavioral tests (Hanlon and Messenger, 1996; Marshall and Messenger, 1996). The exceptions are a deep-sea family including the firefly squid *Watascinia scintillans*, which have three spectral sensitivities, almost certainly co-evolved with their multicolored bioluminescent displays (Michinomae et al., 1994). Recent hypotheses suggesting that the optics of the cephalopod eye may provide chromatic discrimination in some circumstances (Stubbs and Stubbs, 2016a) seem unlikely or of limited use in their normal habitat (Gagnon et al., 2016; Stubbs and Stubbs, 2016b).

Cephalopods are not the only animals capable of color change. Many lizards, particularly the chameleons, as well as many fishes can change both color and pattern (Ramachandran et al., 1996; Stuart-Fox et al., 2006). However, it is the speed with which the skin's appearance can be controlled in cephalopods that is unique. Skin-change capability is deployed by cephalopods in diverse behaviors (Messenger, 2001) and the rapidity of pattern change puts many of these visual displays in a class of their own. With parallels to the pixels on a television screen, cephalopod chromatophores can be coordinated to produce dramatic, dynamic, and rhythmic signals in the form of “flashing” or “strobing,” where fields of chromatophores are opened and closed in synchrony or as moving bands, produced by waves of transiently expanded and contracted chromatophores flowing over the body in a coordinated manner (Packard and Sanders, 1969). We define these display types here collectively as “dynamic patterns.”

The best-known example of a dynamic pattern amongst cephalopods involves a moving pulse or dark band running over the body and arms. This display has been treated by previous authors under the names “passing cloud” or “wandering cloud” (Packard and Sanders, 1969; Hanlon and Messenger, 1988; Mather and Mather, 2004; Adamo et al., 2006; Huffard, 2007). In this manuscript, we avoid using the term “cloud,” as it can imply that this display mimics cloud shadows or dappled light from surface waters playing over the animal, thus presupposing a hitherto unknown function. Our use of the term “dynamic patterns” is distinct from “dynamic camouflage,” a term previously used to describe the general ability of cephalopods to switch between different static color patterns (Hanlon, 2007). It is also distinct from “dynamic mimicry,” a term coined for the mimic octopus, *Thaumoctopus mimicus*, where an individual can fluidly morph between multiple aposematic models (Norman et al., 2001).

Due to their transient nature, the dynamic patterns of cephalopods are seldom observed and rarely recorded in the wild. As a result, very few studies have focused on the form and function of these patterns, other than mentioning them as brief anecdotes. This study attempts to collate evidence of dynamic patterns from a wide variety of sources, including public and private video recordings (presented in Supplementary Information), as well as existing scholarly descriptions, in order to examine the nature, context, and potential functions of dynamic skin patterns across diverse cephalopod taxa.

## Materials and methods

The dynamic displays of 21 species of cephalopod were categorized and described from widely sourced digital video sequences. Ten species were filmed by the authors or colleagues, and the remaining 11 were sourced from the literature or from videos posted by the public online. The diverse range of material made it difficult to obtain standardized quantitative data, and so the study focuses on a qualitative description of the dynamic patterns in question. Where possible, example video has been included in Supplementary Information. Analysis was performed using open-source video playback software (VideoLAN, 2016) and Matlab scripts (Mathworks, 2014). Detailed analysis involved digitizing points on the body of cephalopods or their backgrounds over sequences of video frames using the Matlab analysis script “DigiLite” (Jan Hemmi, University of Western Australia) and plotted graphically using custom scripts (available in Supplementary Information). Digilite is available on request from Jan Hemmi. Alternatively, slimmed down versions of this digitisation script are included as Supplementary Information (dgigas_digitisepoints.m, olaqueus_digitisepoints.m, and latimanus_digitisepoints.m). Measures used to describe each display included temporal frequency or movement speed of the pattern, and the fine-scale behavioral context of the displays.

## Results

Through direct observation, video documentation and externally sourced footage of 21 cephalopod species, we recognize five categories of dynamic skin patterns, with certain species being capable of displaying more than one category: (1) *flashing* (or strobing); (2) *flickering*; (3) *chromatic pulses*; (4) *rhythmic passing waves*; and (5) *multi-directional passing waves*. Cephalopod species known to produce these categories of pattern are treated individually below.

### Flashing patterns

Flashing, or strobing, is the simplest category of dynamic skin pattern. It involves the synchronous activation of skin color or light-emitting components, often across the whole body, resulting in a repeated transition from one skin pattern to another (Hanlon and Messenger, 1996). “Flash behavior” is reported elsewhere in nature, where appendages or plumage are used to rapidly display color, contrast or specific patterns (Cott, 1940; Edmunds, 1974; e.g., deimatic displays: Umbers et al., 2015). In this paper, we deal only with patterns in which the repeated flashing is a clear component of the display. We therefore exclude the myriad examples of cephalopods producing single rapid color changes (sometimes referred to as a flash), often produced as part of anti-predation behavior (e.g., Hanlon and Messenger, 1996; Langridge et al., 2007).

**Figure 2:**
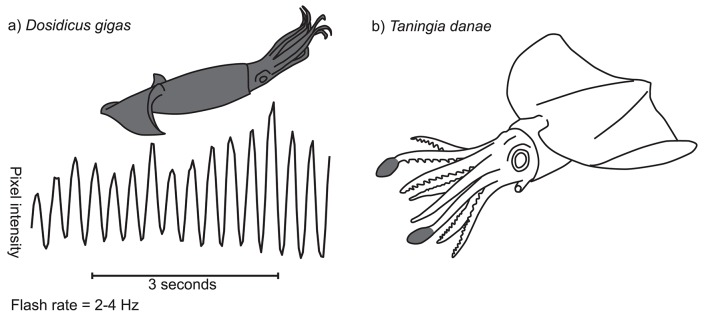
Flashing patterns in two squid species. **(a)** Temporal characteristics of *D. gigas* flashing display. Graph shows, for a single example sequence, pixel intensity for a point on the body on the y-axis (scale not shown) and time along the x-axis. Inset diagram shows in gray the region of the body over which the signal occurs (in this case the whole animal). **(b)** Photophore flashing in *Taningia danae*. Two large bioluminescent organs are located at the distal tips of the dorsolateral arms (gray shaded areas), each of which can be flashed on and off by moving a pigmented cover (redrawn from Richard Ellis).

Flashing patterns have been most reliably documented in the Humbolt squid, *Dosidicus gigas*. Sometimes known as jumbo squid or jumbo flying squid, *D. gigas* is one of the largest and most abundant nektonic species of cephalopod (Nigmatullin et al., 2001). The species inhabits deep ocean areas from the eastern Pacific to the Chilean coast and the Sea of Cortez, where it performs vertical feeding migrations at dusk from the deep to shallower water (Markaida et al., 2005; Gilly et al., 2006; Zeidberg and Robison, 2007; Trueblood et al., 2015). The flashing patterns of *D. gigas* have been recorded and described on numerous occasions, including a recent deployment of the National Geographic “CritterCam” (Marshall et al., 2007; Rosen et al., 2015). Briefly, flashing patterns in this species involve the rapid opening and closing of chromatophores over the whole body in tight synchrony at a frequency of around 2–4 Hz (Figure 2a; Supplementary Video 3.1.1). The pattern tends to occur when other *D. gigas* are nearby, suggesting an intraspecific communication function. The display probably plays a role in courtship as well as during agonistic interactions (Rosen et al., 2015), particularly given the high risk of cannibalization within the species (Markaida and Sosa-Nishizaki, 2003). Rosen et al. (2015) also report a similar dynamic pattern in another large pelagic squid, *Sthenoteuthis oualaniensis*, but data supporting this is as yet unpublished.

Several cephalopod species are known to produce flashing patterns of bioluminescence. The deep sea Dana squid, *Taningia danae*, possesses large occludable photophores on the tips of the dorsolateral pair of arms (Figure 2b; Roper and Vecchione, 1993). These organs have been observed producing brief, synchronous flashes of blue-green light in captured and free-ranging individuals, a display that seems to be associated with attack or escape behavior (Roper and Vecchione, 1993; Kubodera et al., 2007). Similarly, vampire squid, *Vampyroteuthis infernalis*, can dynamically occlude large photophores on the mantle, apparently as an anti-predation strategy (Robison et al., 2003) and the deep sea squid *Octopoteuthis deletron* can flash it's arm-tip photophores in various behavioral contexts (Bush et al., 2009). However, very little is known about the natural ecology of these deep-water species and so further work is needed.

### Flicker patterns

Flicker (or shimmer) patterns involve the non-synchronous activation of skin pattern elements to produce seemingly random shimmering or flickering waves, often across the whole body. Many species show low-level flickering of their skin pattern, possibly due to signal noise in the neuro-muscular control system (e.g., *Idiosepius notoides* example, Supplementary Video 3.2.1; Holmes, 1940; Suzuki et al., 2011). However, several flicker displays with an apparent function have been described in the literature.

The “CritterCam” study of Rosen et al. (2015) recorded multiple instances of flicker patterns in the Humbolt squid, *D. gigas*. They describe the pattern as having a “*noisy wave-like appearance*” across the whole body and observed that it occurs as a “*basal level of chromatophore activity in the absence of flashing*” (Supplementary Information 3.2). The authors go on to suggest that the pattern may act as a form of dynamic crypsis, mimicking the pattern of down-welling light in shallow waters. How the addition of flicker dynamic skin patterns to naturally occurring caustic flicker could have the effect of reducing animal conspicuousness remains to be demonstrated.

A second example of a flicker display can be found in the deep-sea finned octopod *Stauroteuthis syrtensis* and is described from captured and free-ranging specimens by Johnsen et al. (1999). This species possess many small bioluminescent photophores in place of its suckers on the underside of each of its eight arms. These organs can be induced to flash when disturbed, similar to other species. But interestingly, individual photophores were observed blinking on and off asynchronously at about 0.5 to 1 Hz, producing a “*twinkling*” effect. *In situ*, one individual was seen “*spread in the horizontal plane with the mouth upwards*,” leading the authors to suggest that the twinkling photophores may act as light lures for attracting their planktonic crustacean prey, which then become trapped in the arm webs.

### Chromatic pulse

Chromatic pulses are dynamic skin patterns consisting of a single band or spot of color contrast sweeping across part of the animal in a particular direction. Past studies have referred to some of these displays as “*passing cloud*” or “*wandering cloud*,” with some authors proposing that this display mimics the movement of dappled light from surface waters (Packard and Sanders, 1969; Mather and Mather, 2004; Huffard, 2007). In this work, we prefer the term “chromatic pulse” as it does not presuppose the functional mechanism for the display. Data are presented here on six new reports and two previous reports of cephalopod species that employ chromatic pulse displays.

**Figure 3:**
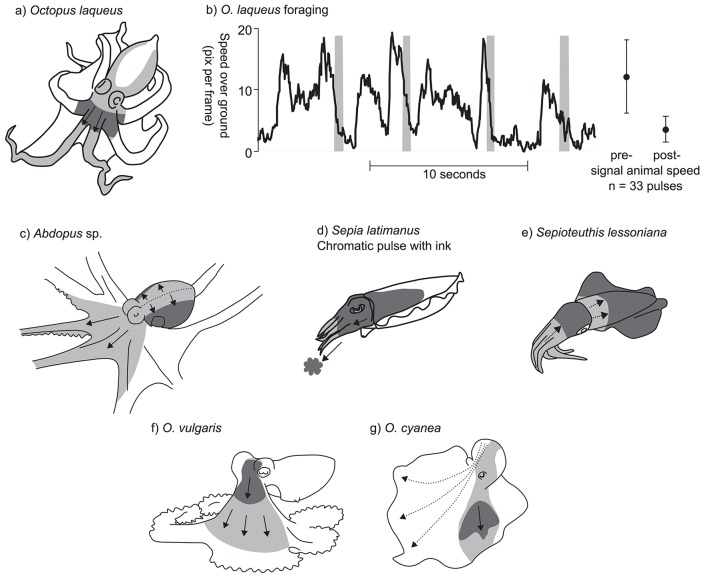
Examples of chromatic pulse patterns. In panels **(a)** and **(c–g)**, light gray indicates the sweep area of the chromatic pulse, and dark gray indicate the position of the pulse at a fixed point in time. **(a)** *Octopus laqueus*. **(b)** Timing of chromatic pulse during *O. laqueus* foraging. Graph presents a subsection of 33 chromatic pulses recorded in a continuous video sequence. Gray bars indicate pulse timing; black line indicates approximate speed over ground of the foraging octopus. Subsequent data points and error bars indicate mean speed ± SD prior to display and after each display is finished. **(c)** *Abdopus* sp. Dotted line indicates line of chromatic pulse origin. **(d)** *S. latimanus* chromatic pulse with coordinated ink jet. **(e)** *Sepioteuthis lessoniana*. **(f)** *O. vulgaris* (redrawn from Packard and Sanders, 1971). **(g)** *O. cyanea* (redrawn from Mather and Mather, 2004). Dotted arrows indicate alternative possible routes for the chromatic patch.

While foraging nocturnally, the tropical octopus, *Octopus laqueus*, produces a chromatic pulse display in which a dark patch passes from the posterior part of the mantle to the arms. Starting at the posterior mantle tip, the pulse diverges to pass bilaterally around the sides of the mantle, and then converges into a single patch at the head. From here the patch continues down to the tips of the dorsal arm pair (Figure 3a). A continuous 140 s video sequence of *O. laqueus* foraging off the Philippines (Supplementary Video 3.3.1) recorded 33 pulses, each lasting 0.55 ± SD 0.11 s, produced at a variable frequency of around 0.25 Hz. The deployment of this display is closely associated with the movement of the animal over the substrate. *O. laqueus* forages by moving in a stop-start pattern across coral rubble, swimming or crawling from one location to the next, then stopping to probe under rubble and into crevices. The chromatic pulse display is synchronized with the “stop” part of the locomotory pattern, each time the animal ceases movement to probe crevices with the arm tips. This is evidenced in the recorded sequence by the animal moving significantly faster (3.5 times) in the moments before each chromatic pulse compared to afterwards (Figure 3b; speed before: 12.1 ± SD 6.0 pixels.s^−1^; speed after: 3.5 ± SD 2.1 pixels.s^−1^; *t*-test: *t* = 8.1, *p* < 0.001). Given the precise behavioral context of the display, we can hypothesize three possible functions. One possibility is that the display acts as a conspicuous warning signal to ward off potential predators as the octopus forages among the coral rubble. The second possibility is that the display acts as a form of motion camouflage during low-light conditions. It may disguise the precise moment when the animal stops moving by continuing a false motion cue in the direction of travel after the animal has stopped. Thirdly, the display may help to flush out prey from the coral rubble, startling them into evasive behavior. It is important to note that this description was based on a single individual in the only known video of *O. laqueous* chromatic pulse patterns, and so further observations and experiments are required to study this in detail.

A very similar dynamic skin pattern was observed in several other octopuses, including an *Abdopus* species, the Caribbean two spot octopus *Octopus hummelincki*, and possibly the Caribbean reef octopus *Octopus briareus*. Observations of an as-yet undescribed species of *Abdopus* were obtained from a series of videos of a single individual moving around shallow rock pools at night near Broome, Western Australia. The chromatic pulse originates along the dorsal midline of the mantle and head, and then spreads laterally across the mantle, extending to the ventral part. The pulse then flows anteriorly along the webs and dorsal arm pair and the dorsal halves of the second arm pair (Figure 3c; Supplementary Video 3.3.2). A single chromatic pulse took 1.2 ± SD 0.07 s to complete (*n* = 13 pulses observed over 93 s from a single individual) with irregular intervals. The pattern was performed while the octopus was raised on its arms, with the mantle held parallel to the substrate. The overall effect is of the chromatic pulse passing from the highest point on the body, down to the lowest part. The chromatic pulses of *O. hummelincki* and *O. briareus* are very similar, but were only observed in individuals housed in personal aquaria (Supplementary Information 3.3; a, b, c, d, e, f, g).

Another variant of the chromatic pulse pattern was observed in the reef-dwelling Broadclub cuttlefish, *Sepia latimanus*. The display was filmed in the daytime on the Great Barrier Reef, Australia (Supplementary Video 3.3.3) during an interaction between a small male and a larger mate-guarding male. The small male assumed a mottled body pattern with a whitened head and arms, and slowly approached the rival male in a direct head-on posture with its arms tucked closely together. The small male then produced a dark blush around the head over a period of about 1 s, then quickly expelled a small cloud of ink, while simultaneously expanding the dark blush down the head and arms (Figure 3d). The latter part of the display was relatively fast (<0.25 s). Given the behavioral context, as well as the nature of the synchrony between the chromatic pulse and the expelled ink, we suggest that this display is used as an aggressive or territorial signal between rival males. It is possible that smaller males incorporate jets of ink to enhance the visual impact of the display.

A similar (although inkless) display has been filmed in the bigfin reef squid *Sepioteuthis lessoniana* (Figure 3e). A video, reportedly from the waters around the United Arab Emirates, shows a day-active *S. lessoniana* performing occasional chromatic pulses, in which the body is first darkened all over, then pulses of white are passed simultaneously from the tips of the arms toward the head, and from the anterior edge of the mantle toward the mantle center (Supplementary Information 3.3). The display appears to be performed in response to the presence of the camera or diver, and may represent a threat signal.

Finally, two other examples of chromatic pulse displays have been previously described in the literature and these show many similarities to those of *O. laqueus, Abdopus* sp., and *O. hummelincki*: Packard and Sanders (1969) described the chromatic pulse display of *O. vulgaris* as “*dark flushes of color that pass as a wave outwards from the head and then fade into the general background mottle*” (Figure 3f). The display tended to be produced during foraging behavior, specifically when pursued crabs stopped moving. These authors proposed that this signal functions to startle the prey into moving (conveying the message “*move you other animal*”). No information on the time of day or ambient lighting conditions under which the display was observed was recorded (Packard and Sanders, 1971; Wells, 1978). Mather and Mather (2004) described the display in *Octopus cyanea* as a “*dark cloud*” moving in a posterior–anterior direction from the mantle, down over the head, and down the arm web (Figure 3g). The duration of the display lasted on average 0.85 s. These authors noted that the exact placement of the moving patches on the body was not fixed, and displays could vary in their patch trajectory. Furthermore, on some occasions, two bilaterally symmetric patches ‘moved” across the body rather than just a single patch on one side. The relative contrast of the dark patch in the *O. cyanea* display was also enhanced by a paling of the surrounding area. The display was only observed during periods of foraging activity within artificial enclosures.

### Rhythmic passing waves

This type of body pattern involves the movement of rhythmic bands of contrast across the skin surface in a single, constant direction. Here, we report four new examples of the display and one from the literature.

**Figure 4:**
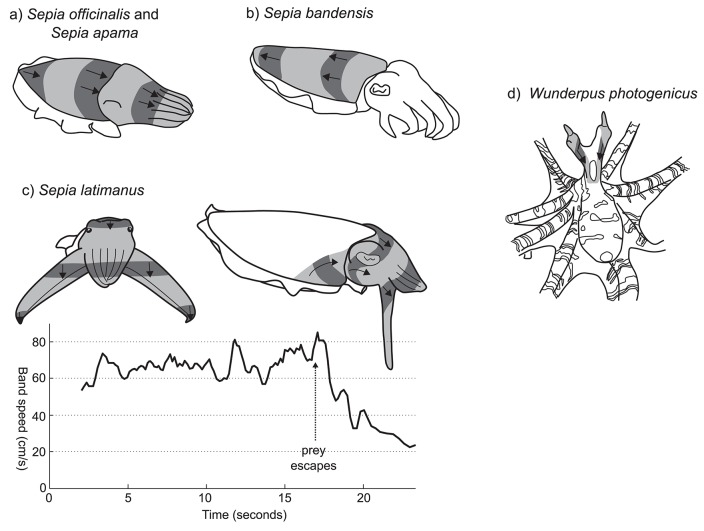
Rhythmic passing wave patterns. **(a)** *S. officinalis* (redrawn from Hanlon and Messenger, 1996) and *S. apama*. **(b)** *S. bandensis*. **(c)** *S. latimanus* front view (left); lateral view (right). Graph shows the approximate change in speed of the banding pattern before and after aborting a predation attempt. Vertical arrow indicates the timing of prey escape. **(d)** *W. photogenicus*. Image shading conventions as per Figure 3.

The unidirectional passing wave display of *Sepia officinalis* has been frequently mentioned in the literature, but not described in detail. One of the earliest descriptions comes from Holmes (1940), “…*the color change seems to result from the passage over the head and arms of waves of contraction and expansion of the chromatophores*.” More detail was provided by Hanlon and Messenger (1988) who described it as, “*a kinetic pattern, lasting only a second or two, characterized by broad transverse bands of chromatophore expansion moving rapidly forward from the posterior mantle tip across the dorsal body surface to the anterior tip of the arms*” (Figure 4a; Supplementary Information 3.4). The stripes move across the body at a frequency of about 1 Hz, and the pattern is often produced by young cuttlefish as they move across a substrate. This display is reported primarily for juvenile cuttlefish during hunting behavior (Holmes, 1940; Hanlon and Messenger, 1996; Adamo et al., 2006), although there is some suggestion that it may also function as a defensive signal in response to approaching predators (Hanlon and Messenger, 1988).

A very similar pattern has been filmed in young giant cuttlefish *Sepia apama* (Supplementary Videos 3.4.1 and 3.4.2). In this species, each wave of contrast takes around 1.5–2 s to pass along the length of the mantle. Although the exact ecological context is not clear, the pattern appears to be produced by camouflaging animals under low light conditions as they locomote in a posterior direction across a substrate.

The dwarf cuttlefish, *Sepia bandensis*, produces a very similar passing wave pattern to *S. officinalis* and *S. apama*, with the difference that the direction of the wave is reversed—from the anterior to the posterior mantle. (Figure 4b; Supplementary Information 3.4; a, b, c, d, e). Unfortunately, little is known about the natural ecology of this species and the contexts in which this pattern may occur.

Perhaps the most striking example of a unidirectional passing wave display in cephalopods is produced by the Broadclub cuttlefish *S. latimanus* during hunting behavior. On sighting a prey item, the cuttlefish will tentatively approach in full camouflage, typically in the “branched coral” pose. Once within 0.5–1 m of its prey it switches to the following, highly conspicuous passing wave pattern (Supplementary Video 3.4.3). The camouflage pattern is replaced by a light uniform whitish color and the first three arm pairs are thrust forwards into a tight cone, while the two ventral arms are splayed outwards and flattened so that the arm/web margin surface is perpendicular to the viewing direction of the prey animal (Figure 4c, left). Moving bands of dark contrast are then generated, passing quickly across the head and anterior mantle at speeds of 40–80 cm/s. These bands originate at the anterior lateral margins of the dorsal mantle, then rotate upwards and anteriorly toward the eye, merging on the dorsal head, then passing down the four arm pairs (Figure 4c, right). When viewed directly from the perspective of the prey animal, the moving bars are oriented so that, despite passing over the unevenly curved head of the cuttlefish and anteriorly projecting arm cone, they appear horizontally straight, moving in a uniformly downwards direction. The speed of the moving bars can be adjusted, and seems to be linked to the behavioral context. The inset graph in Figure 4c presents the downward speed of moving bars during a single prey-approach event recorded on video. Bar speed appears relatively stable around 65 cm/s, while approaching the prey item. But once the prey escapes (arrow at 17 s), the cuttlefish slows the speed of its display to 20–25 cm/s before reverting to static skin patterns. Despite being well-known among divers in Indonesia, as well has having been presented in several high-profile natural history documentaries (Supplementary Information 3.4), we could find no reports of this behavior in the scientific literature.

A final example of a rhythmic passing wave pattern can be found in the octopus *Wunderpus photogenicus*. This Indo-Pacific species produces a rhythmic, unidirectional wave pattern down the eye stalks. Originating at the distal tip of the eye stalks, the chromatic waves pass downwards, over the head and to the junction of the mantle and arm crown, at a frequency of approximately 1–2 Hz (Figure 4d; Supplementary Video 3.4.4; source J. Finn). This display was observed both while the animal was foraging and when within its burrow with only the head protruding, but the ecological function of the pattern is unclear.

### Multidirectional passing wave displays

Multidirectional passing wave displays are similar to the rhythmic passing wave displays described above. However, the moving stripe patterns occur in multiple directions in different parts of the animal's body. We identified several examples of multidirectional passing waves, exclusively within cuttlefish. Most of these patterns are bilaterally symmetric, so that each field is paired across the body midline. We define the number of display fields according to the subunits containing passing waves per side of the mantle. Data is presented here on two-, three-, and five-field dynamic patterns across five cuttlefish species. Some species also display additional rotating bands on the lateral head and arm bases.

**Figure 5:**
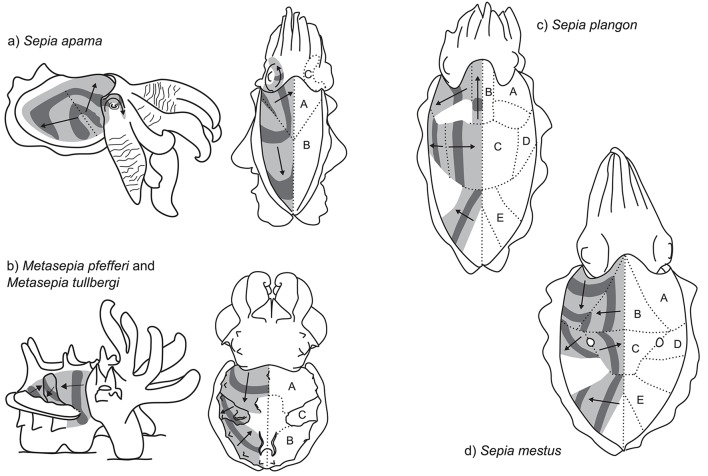
Multidirectional passing wave patterns. **(a)** *Sepia apama*: left—competitive display produced by rival males during the spawning aggregation; right—dorsal view of the two motion fields (without skin stretch). **(b)** The dynamic pattern of the two closely related species, *Metasepia pfefferi* and *M. tullbergi*: left—lateral view; right—dorsal view. **(c)** *S. plangon* (dorsal view). **(d)** *S. mestus* (dorsal view).

One of the most conspicuous examples of this type of dynamic pattern is produced by the giant cuttlefish *S. apama*. During reproductive activity, males can be observed producing a striking dynamic pattern toward competing males (Norman et al., 1999; Hall and Hanlon, 2002). This display is directed toward the recipient cuttlefish during close interactions by tilting the lateral mantle toward the opponent (Figure 5a, left, Supplementary Video 3.5.1). The arms are extended and flared to maximize their visual surface area. The mantle is almost white and repeated dark bands are passed across the nearest lateral half of the mantle surface. The display is relatively slow moving, with each band taking 7.0 ± SD 1.0 s to travel across the mantle and at a low frequency of 0.38 ± SD 0.08 Hz (*n* = 5 individuals). Because the display tends to be viewed by rival males at close range (~10 cm), it occupies a large proportion of the receiver's visual field (visual angle of moving band width ~20° and interval ~30° when viewed from 10 cm). The wave patterns originate along a diagonal line that stretches from the anterio-lateral mantle border with the fin to the midpoint of the medial dorsal mantle (dotted line Figure 5a, left). Waves of contrast are initially propagated synchronously in two different directions, one moving diagonally toward the anterior midpoint of the dorsal mantle (Figure 5a, right, field A), and the other moving diagonally toward the opposite posterior midpoint of the dorsal mantle (Figure 5a, right, field B). As these bands diverge, they remain in contact along the line of divergence, producing the impression of an expanding arch. Occasionally, a third passing wave field is visible on the head in the region of the eye nearest to the rival male (Figure 5a, right, field C). In this region, the waves of contrast commence from behind the eye, rotate over the brow of the eye and onto the base of the arms in synchrony with the other two contributing skin fields. Due to the predominantly lateral orientation and presentation of this display, individuals often contract the skin on the non-signaling side of the mantle to stretch the dynamic display over a larger proportion of the mantle surface (Figures 1b, 5a, left). This display type is typically restricted to a single side of the body. However, in situations where rivals are located on both sides of the displaying male, the dynamic signals can be presented symmetrically on both sides of the body.

The second context for use of this display does not appear to relate to reproduction. Juveniles of this species are occasionally observed amongst moving weed at night, over a light-colored sand substrate, producing a strong, bilaterally-symmetric dynamic pattern almost identical in form and timing to the agonistic display (Supplementary Videos 3.5.2 and 3.5.3). The pattern appears to match the motion of dark sea grass over light sand patches and could represent a form of dynamic camouflage.

Another striking multi-field dynamic pattern is produced by two species of the genus *Metasepia*: the Flamboyant cuttlefish, *Metasepia pfefferi*, and the Paintpot cuttlefish, *Metasepia tullbergi*. As the patterns of these species are very similar, we will describe them together. For a more detailed analysis of *M. pfefferi* see Thomas and MacDonald (2016), and for *M. tullbergi* see Laan et al. (2014). These species inhabit subtidal soft sediments and are typically benthic, employing the fourth arm pair and ambulatory flaps on the ventral surface of the mantle to amble along the seafloor with a quadrupedal walking gait (Roper and Hochberg, 1988). The species hunt by stalking small fishes and crustaceans on the seafloor. When disturbed, the species displays a high-contrast pattern of white, yellow, red, and dark brown. This display often includes a multi-field dynamic pattern (Supplementary Information 3.5; a, b, c, d, e). In some situations, animals produce a two-field display composed of field A—anterior third of the dorsal mantle generating a posteriorly moving vertical bar of contrast—and field B—posterior third of the dorsal mantle generating an anteriorly-moving diagonal bar of contrast (Figure 5b). In some individuals, a third motion field was observed within the central part of the dorsal mantle (field C), consisting of a diagonal band of contrast moving in a ventro-posterior direction (Figure 5b). The combined effect of these three motion fields along with the flamboyant color pattern is a highly conspicuous visual signal. The precise function of the signal remains unknown, although there is some suggestion that it may represent an aposematic signal of toxicity in the flesh of the animal.

In several examples, five or more distinct fields of passing waves could be seen in a single animal. Mourning cuttlefish, *Sepia plangon*, housed in aquaria under low light conditions can be observed producing a striking dynamic pattern (Lee, unpublished data; Supplementary Video 3.5.4). As these displays only occurred during low-light conditions, the exact structure of the signal is difficult to record. However, preliminary examination indicates at least five separate motion fields (Figure 5c, Fields A–E). The movement of the bars of contrast in each field appear to be temporally synchronized with each other, so that in some areas the pattern seems to transfer continuously over into a different field. For example, as the central bar in field C reaches the midline, the patch in field B starts moving toward the head. Then as it reaches the head region, the bar in field A starts moving laterally away from the head. The combined effect of these areas working in synchrony produces the illusion of a continuous movement of contrast, spiraling from the center of the mantle, around toward the head, then laterally to the mantle edge. A similar effect is achieved to the posterior end of the animal, with the temporal correlation of fields C and E. Overall, the display is relatively slow moving, with a repeat frequency of around 0.3–0.5 Hz, although there seems to be variation both within and between individuals.

A similar, but more conspicuous dynamic pattern can be found in the Reaper cuttlefish, *Sepia mestus*. Our data is based on a small number of video clips (Candace McBride, unpublished data; Supplementary Information 3.5; a, b, c, d). In this example, a day-active individual cuttlefish, reacting either to the presence of the observing SCUBA diver or to another individual, positions itself near a small clump of dark weed, then performs a striking dynamic display with five distinct fields of motion (Figure 5d, fields A–E). The movement of the high contrast pattern is combined with the pair of “dorsal mantle white spots” (Packard and Sanders, 1971) in the center of the mantle, from which the pattern in fields C and D emanate. When passing over these spots, the dark bands occlude the white pattern, so that these spots appear to “blink” between black and white. The contrast pattern moves much more quickly than that of *S. plangon*, at a rate of around 1.5 Hz.

### Simultaneous displays

Several species of cephalopod are notable in that they are able to produce more than one distinct type of dynamic skin pattern. Above, we described two different patterns produced by the Australian giant cuttlefish, *S. apama*: the single field passing wave display, in which waves of contrast pass from the posterior end of the mantle toward the head, and the multi-field display, in which waves emanate from a line midway along the mantle (Figures 4a, 5a). We have observed an individual of this species switching quickly between these two patterns, potentially in response to the presence of observing (filming) SCUBA diver. On several occasions the animal expressed both patterns simultaneously for a period of several seconds (Supplementary Video 3.4.1).

## Discussion

Body patterning for camouflage and communication is a well-studied aspect of animal biology (Cott, 1940; Stevens, 2013). Many of these static patterns incorporate movement of the animal to enhance the effect. Unusually, in cephalopods we have the unique opportunity to see how evolution can shape body patterns that incorporate intrinsic dynamic components. All patterns described in this comparative study have several design features in common. Firstly, they tend to be high contrast, involving dark patches moving on light backgrounds. This is most extreme in the *Metasepia* species (Supplementary Information 3.5; a, b, c, d, e) and the essentially “black and white” color of the signals may be linked to the color-blind nature of the cephalopod species described here (Chung and Marshall, 2016). Secondly, they have a relatively narrow range of motion speed or frequency. Several species showed displays outside the typical range of motion speed, including the hunting pattern of the Broadclub cuttlefish, *S. latimanus* (whose display is directed toward prey species rather than conspecifics), and the flashing pattern of *D. gigas* (whose display does not contain intrinsic motion, rather repeated on/off switching between pattern components). It is well-known that motion detectors in animals are contrast and speed sensitive (Borst and Egelhaaf, 1989) and so perhaps, in the absence of color, these design features are likely to be adaptations for increased saliency of the pattern.

Interestingly, the dynamic patterns described herein have striking parallels with some research methods in visual ecology. For example, moving gratings (similar to the Broadclub hunting display) and visual playback of looming patterns (similar to *S. apama* agonistic displays and *Octopus* chromatic pulses) have been used extensively to study the visual capabilities of a wide range of animal species, including cephalopods themselves (e.g., Talbot and Marshall, 2010; Temple et al., 2012). These experimental methods are designed to stimulate the motion detection system of the animal viewing the stimulus, and it seems likely that the natural dynamic displays of cephalopods have evolved for a similar purpose.

### Neural control

The comparative analysis of so many diverse dynamic patterns across the Cephalopoda allows us to expand upon some of their suggested control mechanisms. It has previously been established that motor neurons are responsible for the synchronous control of multiple chromatophores in discrete fields on the skin of cephalopods (Packard, 1974; Froesch-Gaetzi and Froesch, 1977; Packard and Hochberg, 1977). These chromatophore motor units (Boycott, 1961; Dubas and Boyle, 1985) are controlled centrally from the chromatophore lobes and stellate ganglion (Young, 1976; Dubas et al., 1986; Williamson and Chrachri, 2004). How these discrete, yet overlapping skin fields are coordinated to elicit specific patterns remains a complex and unsolved problem.

One possible mechanism for generating the dynamic patterns of cephalopods is through endogenous processes in the skin, otherwise termed “myogenic” control. The muscular units responsible for expanding individual chromatophore sacs are known to be electrically coupled to neighboring units (Florey, 1969; Florey and Kriebel, 1969; Reed, 1995) and, under certain experimental conditions, randomly moving passing waves of expanding and contracting chromatophores can be induced in cephalopod skin in the absence of any central control (Sanders and Young, 1974; Messenger, 2001). However, it seems unlikely that this mechanism could be behind the complex and highly controlled dynamic patterns reported here.

Through a detailed analysis of the complex dynamic pattern of *M. tullbergi*, Laan et al. (2014) propose an alternative neural control system originating from the central nervous system. They suggest that passing wave patterns could be controlled via a set of oscillatory neurons analogous to the central pacemakers governing rhythmic locomotory movements. Indeed, control networks for skin chromatophores and swimming fin motor neurons are known to coexist in parts of the cuttlefish brain and stimulation of these areas can result in both patterning and locomotory behavior (Messenger, 2001; Osorio, 2014). This kind of central control could generate dynamic wave patterns in single skin fields, and, most interestingly, could be applied to multiple skin fields resulting in the synchronous activation of different pattern units, such as those in the *Metasepia* species (Laan et al., 2014). Furthermore, central control would permit the speed of passing waves to be adjusted depending on behavioral context, and for different dynamic and/or static patterns to be co-expressed (e.g., *S. latimanus, S. apama*, and *M. tullbergi*; Laan et al., 2014).

As an interesting addendum to this, it must be noted that the octopods recorded in our study do not produce rhythmic passing wave patterns (with the exception of the eyestalk waves of *W. photogenicus*), rather single, non-rhythmic, chromatic pulses. It seems no coincidence that these species also lack the rhythmically controlled lateral swimming fin of *Sepia*. Instead, perhaps the chromatic pulse control system has its origins in different locomotory motor circuits, such as those governing mantle contraction for jetting behavior. Indeed, in some video sequences it appears that mantle contraction and chromatic pulses occur in synchrony (e.g., *Sepioteuthis lessoniana*, Supplementary Information 3.3; and the chromatic pulse/ink jet combination of *S. latimanus*, Supplementary Video 3.3.3) adding weight to this suggestion.

### Dynamic displays across diverse taxa

The dynamic skin patterns described here occur across a wide diversity of cephalopod groups, with some forms reported across six cephalopod orders—squids (Teuthida), cuttlefishes and pygmy squids (Sepioidea), finned octopods (Cirrata), finless octopods (Incirrata), and vampires (Vampyromorpha).

The breakdown of forms of display by taxonomic group reveals some patterns. Strobing and flashing are primarily associated with squids, in the form of strobing in the oegopsid Humbolt squid, and parallels in light flashing in other oegopsid squids and iridescent flashing in loliginid squids.

Chromatic pulses appear to be the domain of benthic octopuses and cuttlefishes. The extent of these displays across the more than 350 octopod species and more than 100 cuttlefish species that exist is virtually unknown as the vast majority have not been observed live. Of the more than 70 shallow-water octopus species observed by two authors of the current study (M. Norman and J. Finn), such displays appear restricted to a small subset and primarily occurred in diurnal species. The pulse displays of *O. laqueus* while night hunting appears to be an exception. Dynamic displays were never observed for a number of common shallow-water genera, such as the night-active genus *Callistoctopus* nor the predominantly crepuscular genus *Amphioctopus*.

For cuttlefishes, the presence of such displays across distinct genera (*Sepia* and *Metasepia*) and multiple species suggest that this form of the display may occur more widely in the group. Due to their excellent crypsis and sudden flight from divers, observations of natural behaviors in shallow-water cuttlefishes are rare. Many species also occur beyond diving depths (e.g., >30 m) and are yet to be observed live.

By gender, dynamic displays are part of the repertoire of males in courtship displays for a number of cuttlefish species, particularly for the Australian giant cuttlefish, *S. apama*, where dynamic displays were not observed in females of the species in breeding aggregations (Norman et al., 1999). The more solitary octopuses lack elaborate courtship displays and we know of no evidence of gender -specific dynamic displays in this group.

Dynamic displays used as camouflage and/or as a component of hunting behaviors (e.g., *S. latimanus*) were observed in both juvenile and adult cuttlefishes and may represent a basal capacity from which reproductive display capacities are likely to have evolved.

### Function of displays

In many of the examples described in this paper, the precise behavioral function of the display is unknown or poorly studied. Based on the context in which the pattern was observed we can make some educated guesses as to the broad functional category that they fall into. In general, the dynamic patterns could be described as either fulfilling the function of (A) deceiving or (B) communicating with the target viewer, with most of the examples in this study falling into the latter.

#### (A) deception

Using dynamic components of body patterns to deceive intended viewers is a novel area of study that has receive little attention in the scientific literature. Here we described the display of several species that seem to do just this.

The clearest example is the hunting display of the Broadclub cuttlefish *S. latimanus* (Figure 4c, Supplementary Video 3.4.3). This pattern is directed toward prey during the final moments of approach, and its highly conspicuous and unusual appearance has led many divers to use terms such as “mesmerizing” or “hypnotizing.” Whether or not the pattern alters the behavior of the intended prey in some way remains to be demonstrated, but it would seem unlikely to have evolved this hunting strategy without some increase in predation success. One possibility is that the downward trajectory of the passing waves provides an overlaying motion cue that masks the expanding motion of the cuttlefish outline as it approaches, as a form of motion camouflage. A similar effect has been recorded from motion-detecting neurons in locusts, in which the sensitivity to localized looming cues is inhibited by broad-field motion cues (Simmons and Rind, 1997). Another hypothesis is that the passing wave motion is so unusual and beyond the standard repertoire of natural motion patterns experienced by the prey item that it causes a confused delay in the escape response. A final hypothesis is that the pattern may induce an optokinetic flow-field response in the prey that alters the position or posture of the animal, centering it between the pulsating arms and facilitating a tentacle strike from the cuttlefish. Further research is clearly necessary to determine the precise mechanism of action.

Another dynamic pattern that is directed toward prey is the chromatic pulse of *Octopus vulgaris* and *O. cyanea* (Packard and Hochberg, 1977; Mather and Mather, 2004). This display may deceive the prey item by simulating an approaching object, thus inducing the prey animal to move, presumably to facilitate capture in some way.

A further example of a dynamic display with a potentially deceptive function is the expanding waves of the juvenile giant cuttlefish, *S. apama*. Animals have been observed producing this usually conspicuous display while camouflaging among seaweed moving in the swell (Supplementary Videos 3.5.2 and 3.5.3). The motion characteristics of the display are not unlike the motion of the surrounding weed, leading us to conclude that this dynamic pattern is being produced to blend in to the movement of the environment. Interestingly, this behavior, as well as that of the Mourning cuttlefish, *S. plangon* (Figure 5c, Supplementary Video 3.5.4), was only observed at night or under low light conditions in aquaria, suggesting that it may be less effective during the daytime, when the motion may instead render the animal more conspicuous.

#### (B) communication

Other dynamic patterns are produced during close interactions with conspecifics, implying a communication function. One of the clearest examples of this is the male-male threat display of *S. apama* performed during mate guarding (Figures 5a; Supplementary Video 3.5.1). This slow-moving, expanding display has several features that may enhance the signal's function. Firstly, the expanding motion cue originates from a lateral position on the anterior mantle edge, close to the location of the nearest eye of the observing rival. Although it is difficult to film the display from the precise position of the observing animal, it is possible to imagine that this expanding cue may appear intimidating, possibly even simulating the expanding motion of an approaching rival. The display is further enhanced by contracting the skin on the lateral half of the mantle away from the rival, thereby stretching the signaling skin field across the midline to substantially increase the visual angle subtended by the display. Whether through just being a large, confusing, moving area or a more directed flow-field pattern giving an illusion of self-motion, the end result of intimidating or driving a rival away appears to be the same.

Another clear example of a dynamic pattern used for communication is the chromatic flashing display used by Humbolt squid, *D. gigas*, during group foraging or mating behavior. Rosen et al. (2015) observed individuals of this species performing the display in the presence of other displaying conspecifics. Given that this species is known to be highly cannibalistic (Markaida et al., 2008), presumably one of the main functions of the display is as a warning or identification signal to conspecifics in the area.

The chromatic pulse exhibited by small male *S. latimanus*, in combination with a jet of ink, represents another clear example of a directed communication signal (Figure 3d; Supplementary Video 3.3.3). We observed the display being performed repeatedly by a small male during full daylight as it tentatively approached a larger rival male, suggesting an antagonistic or bluffing signal. It is tempting to think that the coordination of chromatic pulse and ink jet has the overall effect of extending the motion cue of the moving dark patch beyond the borders of the animal's skin, as a sort of “bluff” signal. However, further research is needed to demonstrate this clearly.

Finally, a different function of a dynamic pattern is likely exhibited by the *Metasepia* species *M. pfefferi* and *M. tullbergi* (Figure 5b; Supplementary Information 3.5; a, b, c, d, e). These bold and striking patterns are produced strongly when the animal is startled by a diver or potential predator, showing clear parallels with other types of warning coloration (Cott, 1940; Ruxton et al., 2004). These species are slow moving and usually found walking with a quadrupedal gait across the sea floor. They are without obvious weaponry, so it is tempting to conclude that the warning display represents a form of aposematic signal. However, no toxicological study of the flesh of the animal has been published to date, so further study is required.

## Conclusion

Cephalopods and their dazzling array of visual representations and behaviors continue to fascinate human observers. Given the generally shy nature of this animal group, the complexity of visual signaling reported here is likely to be a fraction of the potential behaviors yet to be discovered. As a result, the role of “pattern motion” in cephalopod visual displays remains a largely unexplored area of research and warrants greater investigation in both laboratory and field settings. In particular, further work is essential for cataloguing the displays and the fine-scale behavioral context in which they are performed in the natural environment. Furthermore, the identification of species that can be elicited to produce the displays in controlled lab environments would allow an experimental approach to investigate the form and function of these enigmatic patterns.

## Ethics statement

This study is based purely on observations of animals behaving naturally.

## Author contributions

Study conceived by MH and NM. Data provided by MH, MN, JF, and WC. Manuscript written by MH. Comments and edits were contributed by all authors.

### Conflict of interest statement

The authors declare that the research was conducted in the absence of any commercial or financial relationships that could be construed as a potential conflict of interest.
