# Supplementary material for: Dynamic Skin Patterns in Cephalopods
Source: Front Physiol. 2017 Jun 19;8:393. doi: 10.3389/fphys.2017.00393 (PMC5474490; doi:10.3389/fphys.2017.00393)
Supplement: Supplementary file 1 [file Presentation1.PDF]

Supplementary Material

Dynamic skin patterns in cephalopods: a comparative study

Martin J How*, Mark D Norman, Julian Finn, Wen-Sung Chung, and N Justin Marshall

*** Correspondence:** Martin J How: m.how@bristol.ac.uk

This document references the supplementary video clips and link to online content describing the dynamic skin patterns of cephalopods. Section numbering reflects the position in the main manuscript text.

3.1 Flashing patterns

Dosidicus gigas

Supplementary video 3.1.1. A group of flashing Humbolt squid attracted to a baited lure. Source: Julian Finn, Sea of Cortez, 2003. https://dx.doi.org/10.6084/m9.figshare.3841182.v1.

Supplementary video from Rosen et al (2015):
http://movie.biologists.com/video/10.1242/jeb.114157/video-1 and
http://movie.biologists.com/video/10.1242/jeb.114157/video-2

BBC Motion clip of feeding jumbo squid exhibiting strong flashing displays:
http://www.arkive.org/humboldt-squid/dosidicus-gigas/video-00.html

Taningia danae

Supplementary video from Kubodera et al (2007):
http://rspb.royalsocietypublishing.org/content/274/1613/1029.figures-only

NOAA Okeanos Explorer Expedition log:
http://oceanexplorer.noaa.gov/okeanos/explorations/ex1504/dailyupdates/media/ex1504-squid-0924.html

3.2 Flicker patterns

Supplementary video 3.2.1. Source: Wen-Sung Chung showing constant flickering in the chromatophores of *Idiosepius notoides*. https://dx.doi.org/10.6084/m9.figshare.3859014.v1.

Sequence from Roger Hanlon's Lab showing constant flickering in the chromatophores of *Loligo pealeii*: https://www.youtube.com/watch?v=b5F-7Bu8YBQ

Dosidicus gigas

Supplementary video from Rosen et al 2015:
http://movie.biologists.com/video/10.1242/jeb.114157/video-3

3.3 Chromatic pulse

Octopus laqueus

Supplementary video 3.3.1. Around 1.5m depth during nocturnal foraging. Source: Matteo Guardini, Philippines, 2009. https://dx.doi.org/10.6084/m9.figshare.3859020.v1.

***Abdopus* sp.**

Supplementary video 3.3.2. Filmed in a rock pool near Broome, Northwest Australia. Source: Julian Finn and Mark Norman, 1999. https://dx.doi.org/10.6084/m9.figshare.3859029.v1.

Octopus hummelincki

Animal housed in a personal aquarium, posted on YouTube by user ‘D Whatley’:
https://www.youtube.com/watch?v=4RY63hU--co and
https://www.youtube.com/watch?v=_2fsxMvafbU

Octopus briareus

Animal housed in a personal aquarium, posted on YouTube by user ‘D Whatley’ (2010-2012):
https://www.youtube.com/watch?v=42U_3E8-0NM,
https://www.youtube.com/watch?v=HvVbWEYw9kw,
https://www.youtube.com/watch?v=lmHAJcOxrhg, and
https://www.youtube.com/watch?v=8kGudfUw9pw; and by user ‘Michael Nevelyn’ (2009):
https://www.youtube.com/watch?v=7lHe4XK3qK4

Sepia latimanus

Supplementary video 3.3.3. Small male confronting a larger male produces a chromatic pulse with synchronized ink jet. Source: Mark Norman, Great Barrier Reef, Australia, 1998.
https://dx.doi.org/10.6084/m9.figshare.3859041.v1.

Sepioteuthis lessoniana

Videos of free-ranging animal filmed in Mussandam, Oman, posted on YouTube by user ‘yshimy’ (2012): https://www.youtube.com/watch?v=adoy9ymRmc0

3.4 Passing waves

Sepia officinalis

Juvenile filmed in Baleal, Portugal, posted on YouTube by user ‘Joao Pedro Silva’ (2009): http://www.youtube.com/watch?v=tu7TaHb4v74

Sepia apama

Supplementary video 3.4.1. Adult performing simultaneous passing waves and multi-field passing waves. Source: Mark Norman, Port Victoria, 1998. https://dx.doi.org/10.6084/m9.figshare.3859050.v1.

Supplementary video 3.4.2. Presumably *Sepia apama*, although not positively identified. Filmed off Rockingham, Western Australia by Mark Norman in 2007. https://dx.doi.org/10.6084/m9.figshare.3859059.v1.

Sepia bandensis

Short video produced by KQED during a visit to the California Academy of Science (2010): https://ww2.kqed.org/quest/2010/04/27/producers-notes-cool-critters-dwarf-cuttlefish/

YouTube videos of animals housed at the Vancouver Aquarium, posted by user ‘Camille Faubert’ (2013): https://www.youtube.com/watch?v=5c_YBVt1Zi0; at seattle aquarium, posted by user ‘Chris Sully’ (2010): https://www.youtube.com/watch?v=cy9dVKx5ei8; and in personal aquaria posted by user ‘Daniel Pon’ (2008): https://www.youtube.com/watch?v=M5bv_kn0Gkc; and ‘spinycheek’ (2009): https://www.youtube.com/watch?v=3Jk7BWoXFng

Sepia latimanus

Supplementary video 3.4.3. Adult hunting a crab (crab provided by the camera man). Source: Julian Finn, Lembeh Strait, North Sulawesi 2006. https://dx.doi.org/10.6084/m9.figshare.3859062.v1.

The behavior also appears in the following natural history documentaries: ‘The Triumph of Life’, Green Umbrella productions 1999. www.green-umbrella.co.uk; and ‘Cuttlefish: The Brainy Bunch’, Kaufmann productions 2007. www.kaufmannproductions.com.

Wunderpus photogenicus

Supplementary video 3.4.4. Passing waves near the eyes of *Wunderpus*. Source: Julian Finn, 1998, Lembeh Strait, North Sulawesi. https://dx.doi.org/10.6084/m9.figshare.3859065.v1.

3.5 Multi-field

Sepia apama

Supplementary video 3.5.1. Adult male producing mate-guarding threat display. Martin How, South Australia, 2008. https://dx.doi.org/10.6084/m9.figshare.3859068.v1.

Supplementary video 3.5.2 and 3.5.3. Camouflaging juvenile producing two-field passing wave pattern. Source: Mark Norman, Albany, Western Australia 2007, and Chris Merritt, Rottnest Island, Western Australia 2008. https://dx.doi.org/10.6084/m9.figshare.3859071.v1, https://dx.doi.org/10.6084/m9.figshare.3859074.v1.

Metasepia pfefferi

Supplementary videos available with the study of Thomas and MacDonald (2016). https://peerj.com/articles/2035/#supplemental-information

YouTube videos of animals in the wild at Lembeh Straight, Indonesia, posted by user 'Eunjae Im' (2014): https://www.youtube.com/watch?v=U11x5NZ1TAw; and off Kapalai Island, Sabah, posted by user 'Mohammad Azri' (2007): https://www.youtube.com/watch?v=tHnm4I4RBMo

Metasepia tullbergi

Supplementary video available with the study of Laan et al. (2014). http://www.cell.com/cms/attachment/2016900635/2037386554/mmc2.mp4

YouTube videos of animals in the wild posted by user 'Japan Marine Club' (2013): https://www.youtube.com/watch?v=yiPgWFNaKHs

Sepia plangon

Supplementary video 3.5.4. Adult producing a multi-field passing wave in near-total darkness (filmed using red light only). Source: Yi-Hsin Lee, Stradbroke Island, Australia 2011. https://dx.doi.org/10.6084/m9.figshare.3859080.v1.

Sepia mestus

Four sequences filmed in Australia posted on Shutterstock by user 'Undersea': http://www.shutterstock.com/video/clip-2532908-stock-footage-reaper-cuttlefish-sepia-mestus-changing-colour-underwater-in-australia.html, http://www.shutterstock.com/en/video/clip-2532890-stock-footage-reaper-cuttlefish-sepia-mestus-changing-colour-underwater-in-australia.html, http://www.shutterstock.com/video/clip-18616433-stock-footage-reaper-cuttlefish-changing-colour-sepia-mestus-hd-up.html, and http://www.shutterstock.com/video/clip-18616418-stock-footage-reaper-cuttlefish-changing-colour-sepia-mestus-hd-up.html

References

Kubodera, T., Koyama, Y., and Mori, K. (2007). Observations of wild hunting behaviour and bioluminescence of a large deep-sea, eight-armed squid, *Taningia danae*. *Proceedings of the Royal Society B: Biological Sciences* 274(1613)**,** 1029-1034. doi: 10.1098/rspb.2006.0236.

Laan, A., Gutnick, T., Kuba, Michael J., and Laurent, G. (2014). Behavioral analysis of cuttlefish traveling waves and its implications for neural control. *Current Biology* 24(15)**,** 1737-1742. doi: 10.1016/j.cub.2014.06.027.

Rosen, H., Gilly, W., Bell, L., Abernathy, K., and Marshall, G. (2015). Chromogenic behaviors of the Humboldt squid (*Dosidicus gigas*) studied in situ with an animal-borne video package. *Journal of Experimental Biology* 218(2)**,** 265-275.

Thomas, A., and MacDonald, C. (2016). Investigating body patterning in aquarium-raised flamboyant cuttlefish (*Metasepia pfefferi*). *PeerJ* 4**,** e2035. doi: 10.7717/peerj.2035.
