# Supplementary figures and images for: Dynamic Skin Patterns in Cephalopods
Source: Front Physiol. 2017 Jun 19;8:393. doi: 10.3389/fphys.2017.00393 (PMC5474490; doi:10.3389/fphys.2017.00393)

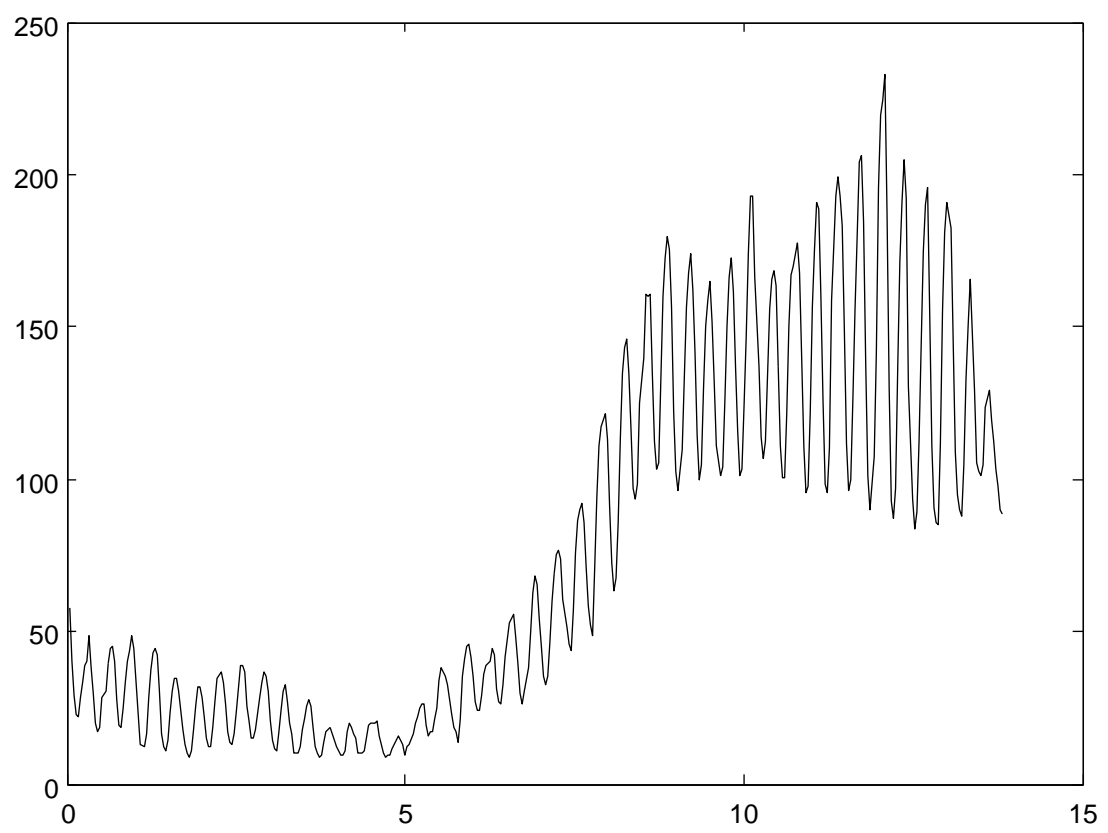

Supplement: Supplementary file 3 [file DataSheet2.ZIP › outputgraphs/Dgigas_tline.pdf]

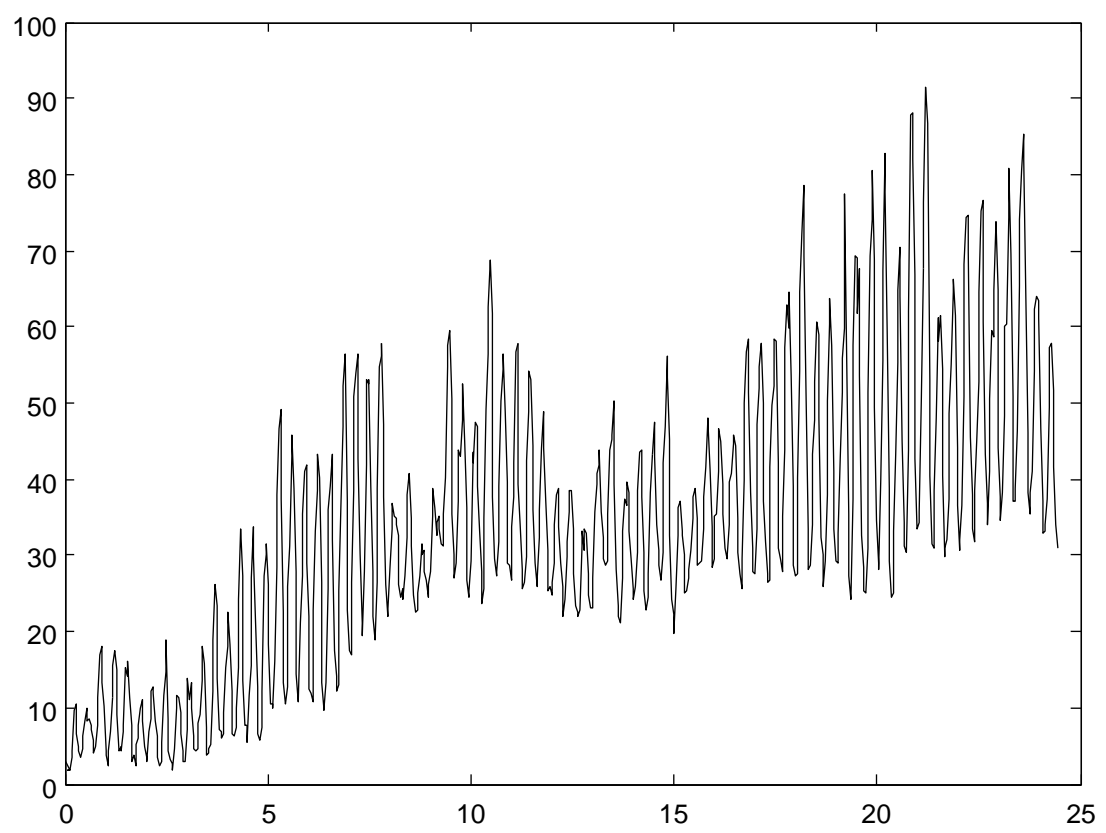

Supplement: Supplementary file 3 [file DataSheet2.ZIP › outputgraphs/Dgigas_tline2.pdf]

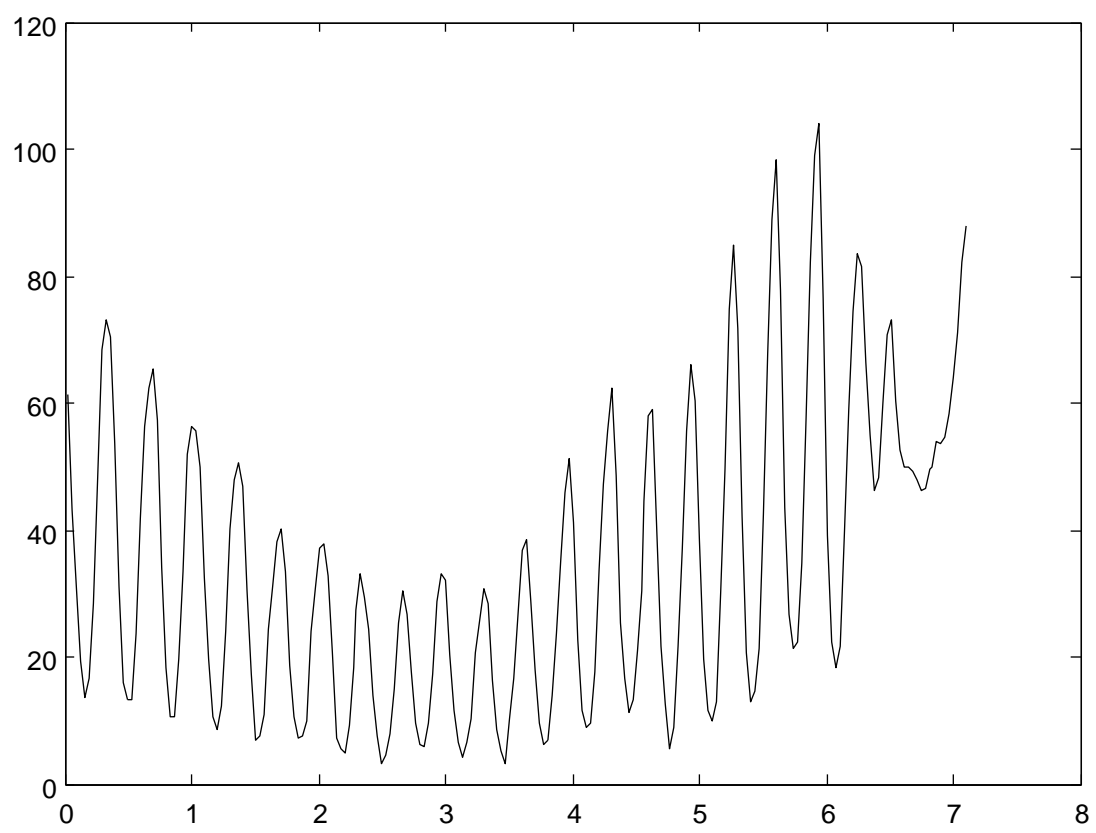

Supplement: Supplementary file 3 [file DataSheet2.ZIP › outputgraphs/Dgigas_tline3.pdf]

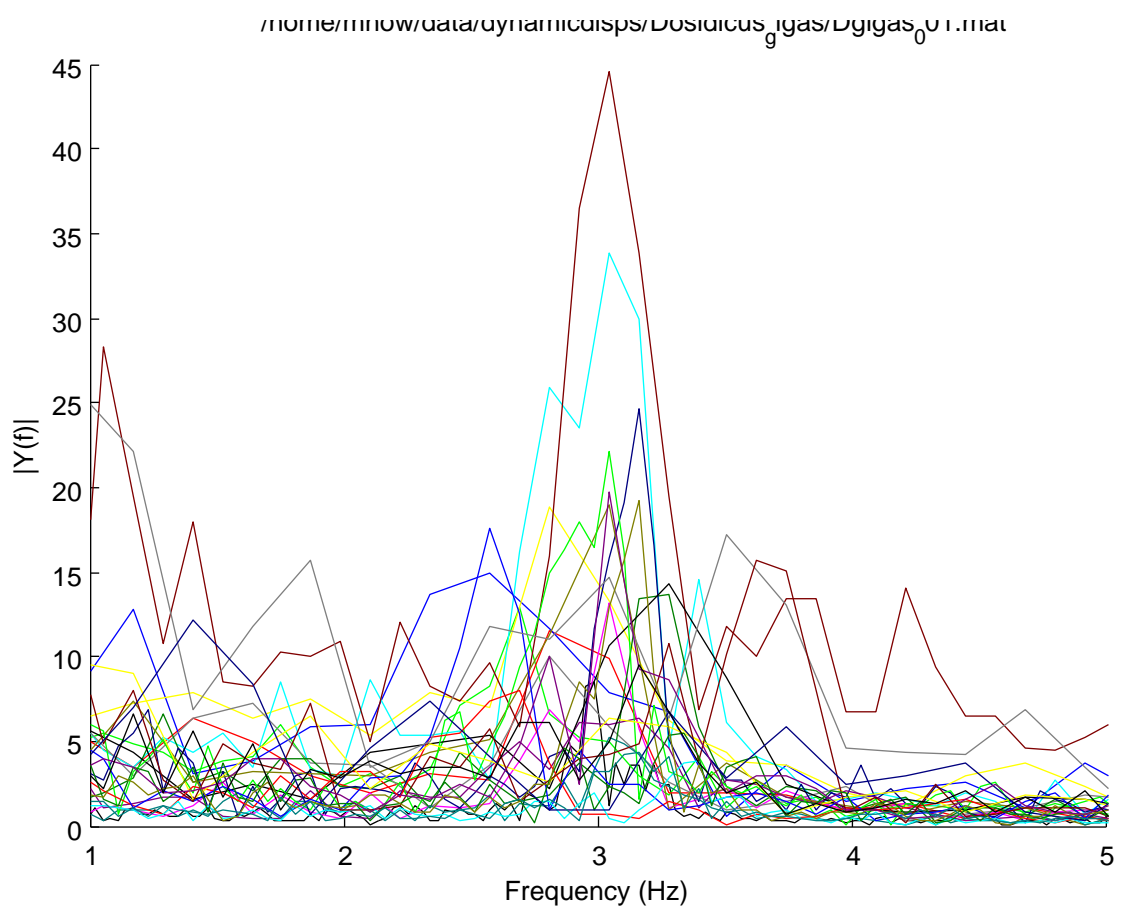

Supplement: Supplementary file 3 [file DataSheet2.ZIP › outputgraphs/Dgigas_tline4.pdf]

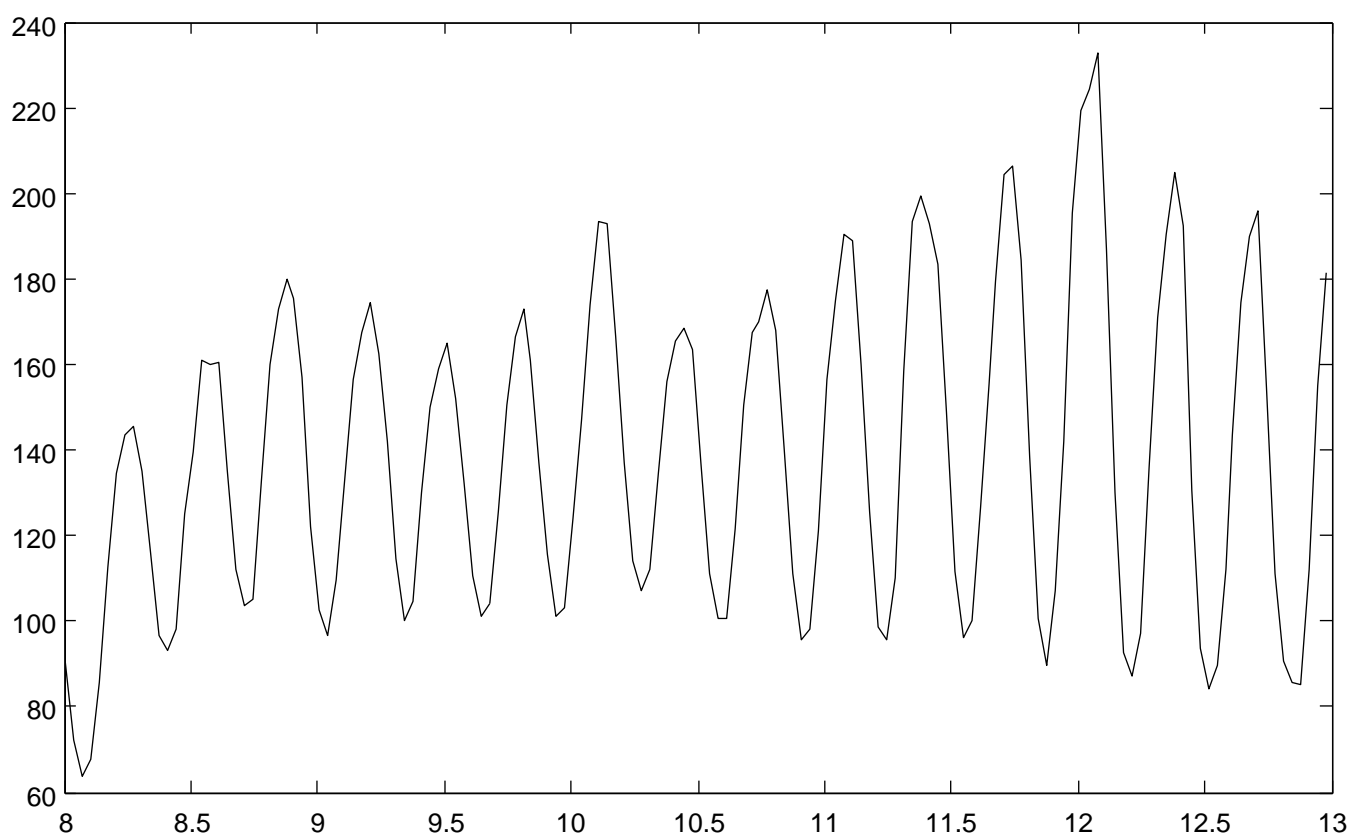

Supplement: Supplementary file 3 [file DataSheet2.ZIP › outputgraphs/Dgigas_tline5.pdf]

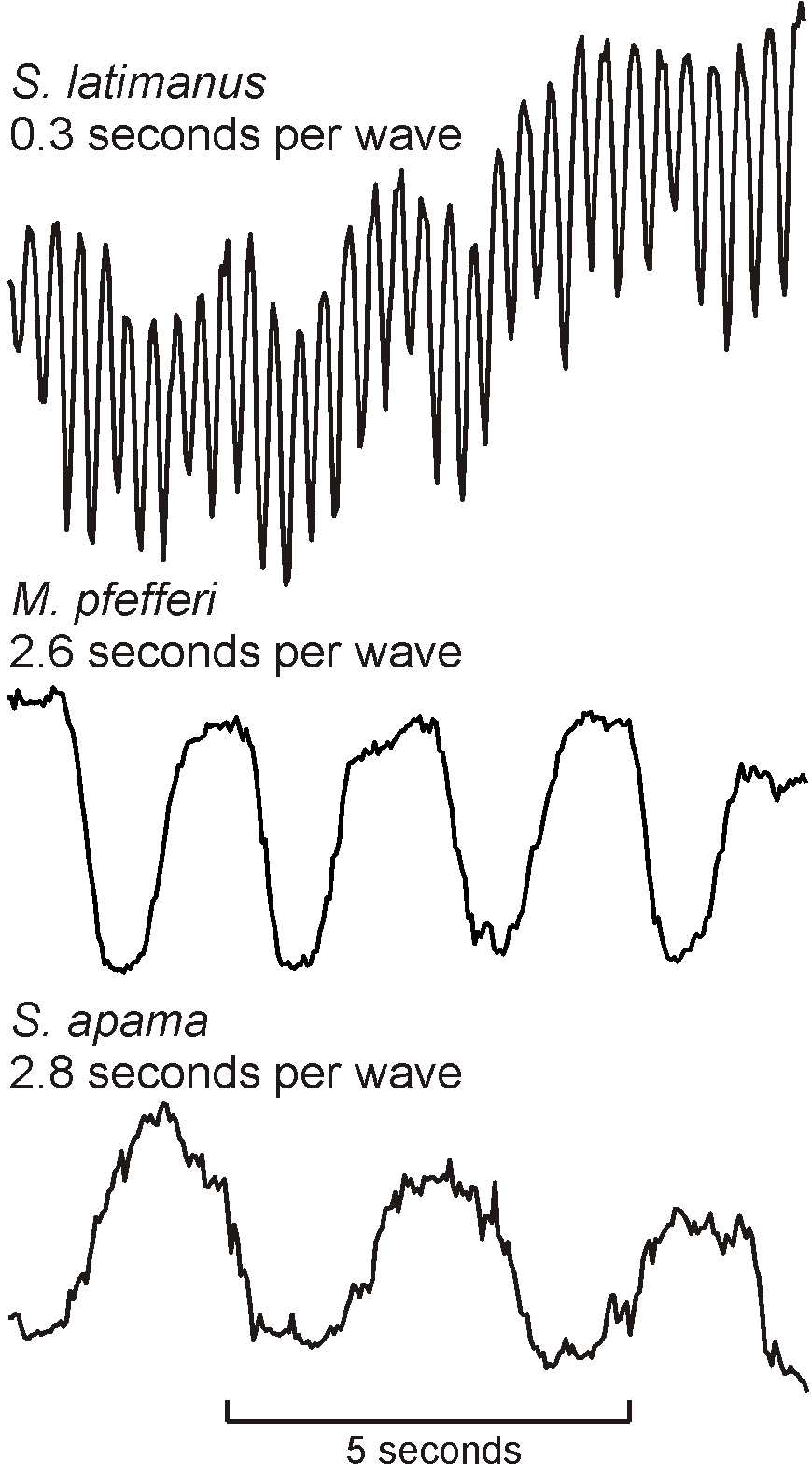

Supplement: Supplementary file 3 [file DataSheet2.ZIP › outputgraphs/frequencies.tif]

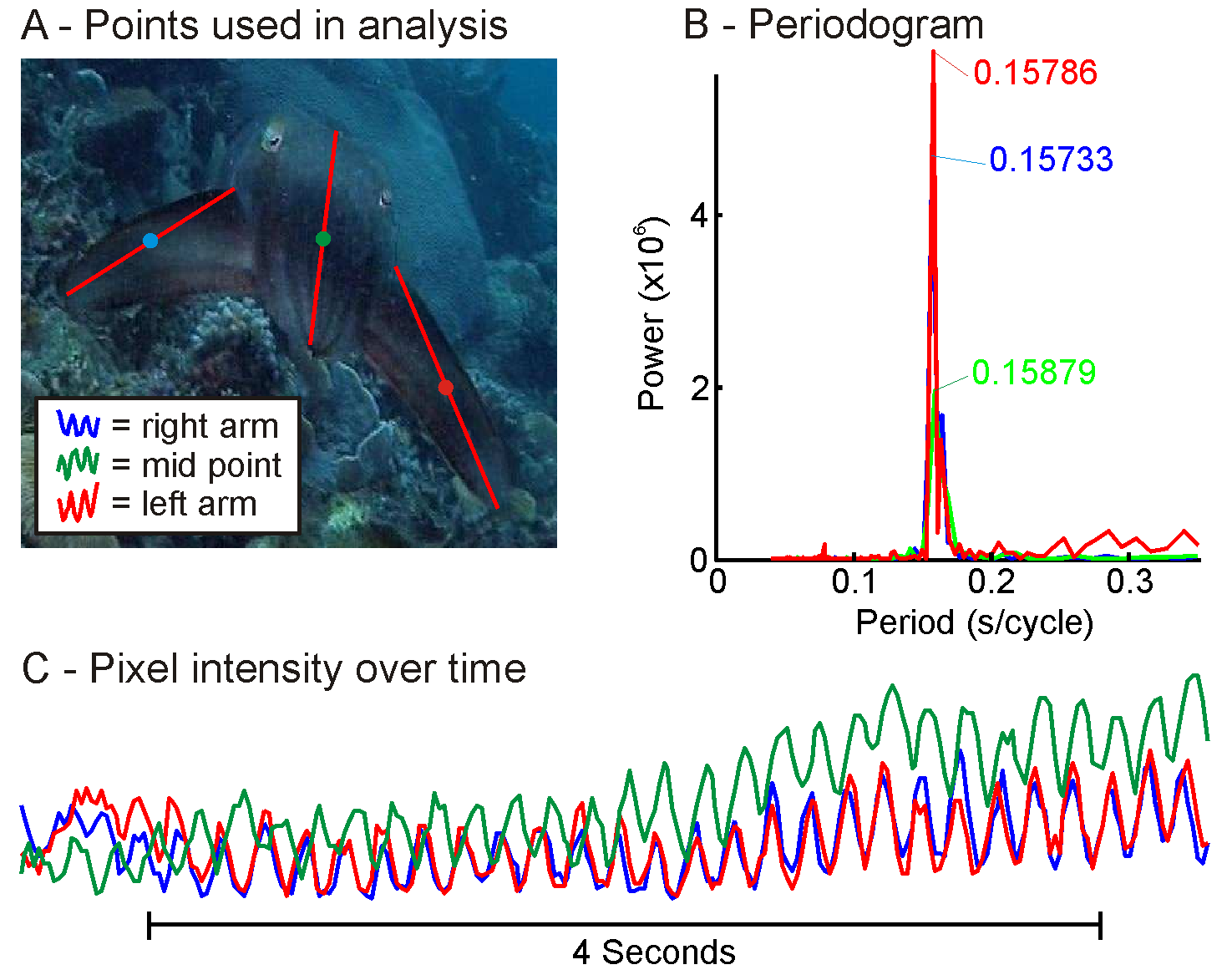

Supplement: Supplementary file 3 [file DataSheet2.ZIP › outputgraphs/latimanus periodicity.tif]

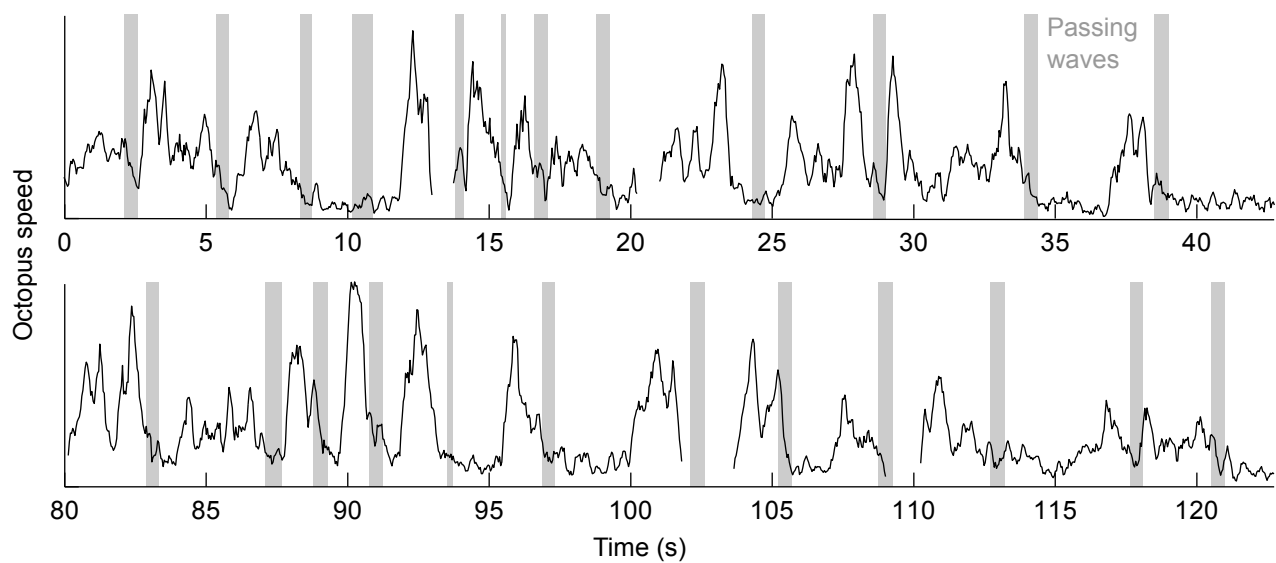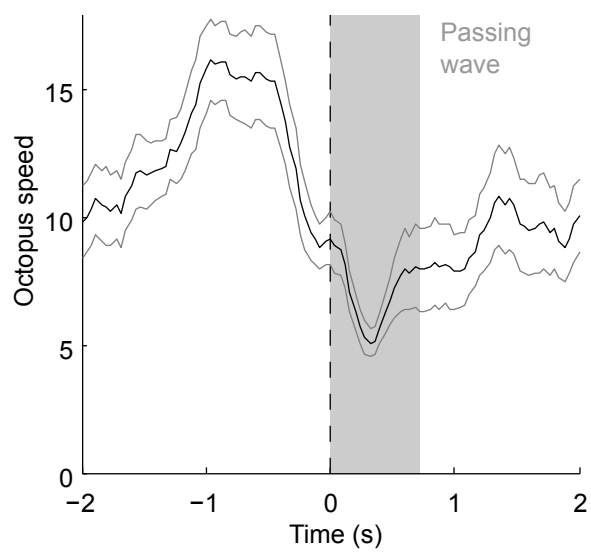

Supplement: Supplementary file 3 [file DataSheet2.ZIP › outputgraphs/Olaqueus02.pdf]

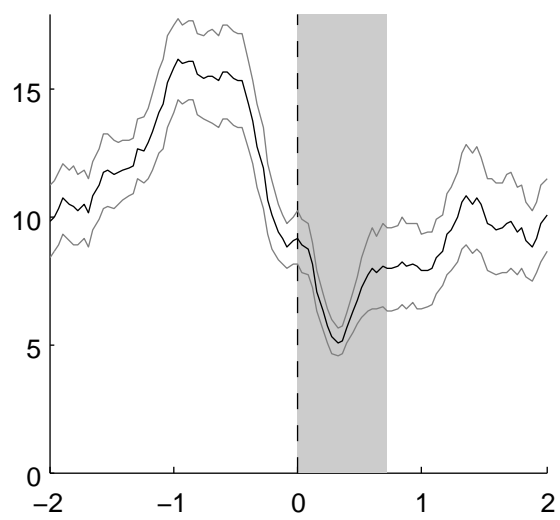

Supplement: Supplementary file 3 [file DataSheet2.ZIP › outputgraphs/Olaqueus02b.pdf]

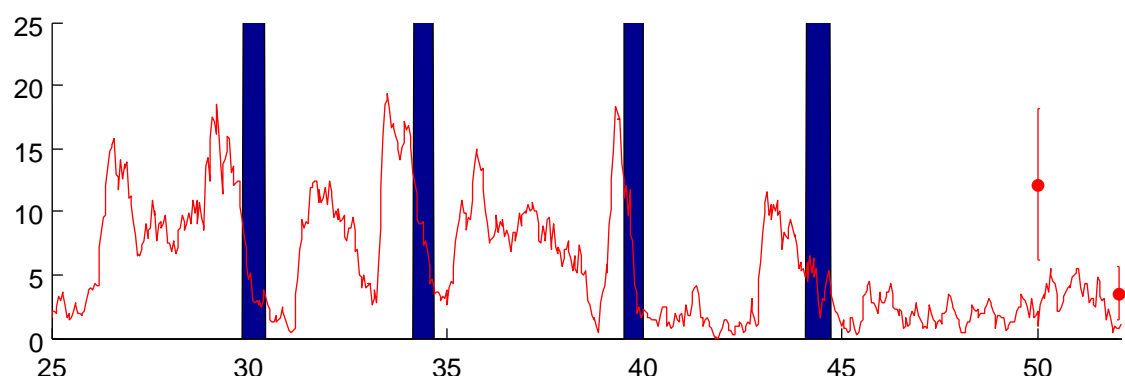

Supplement: Supplementary file 3 [file DataSheet2.ZIP › outputgraphs/Olaqueus_new.pdf]

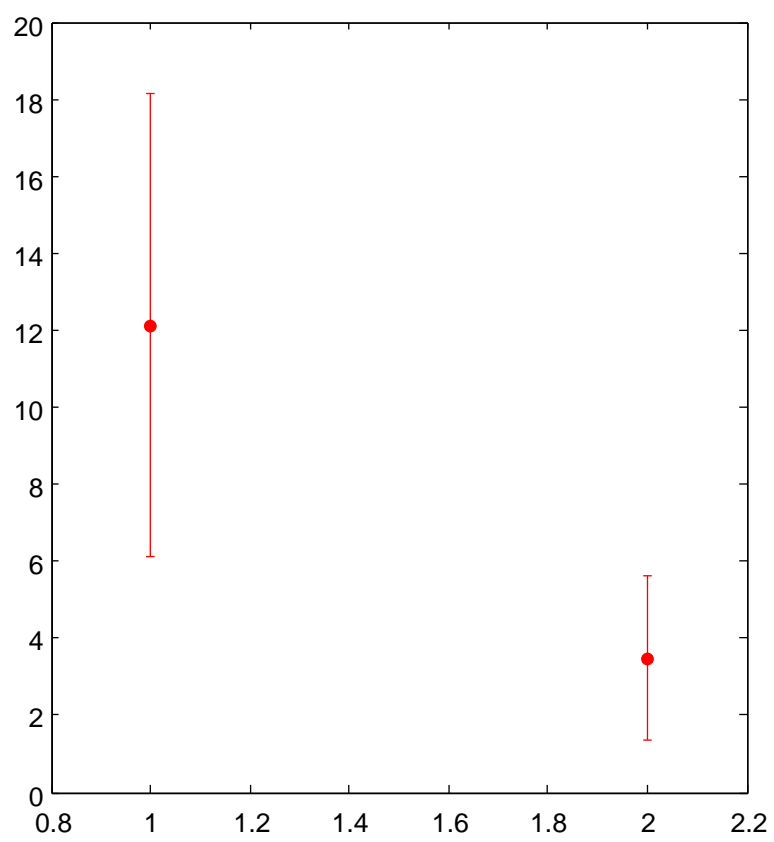

Supplement: Supplementary file 3 [file DataSheet2.ZIP › outputgraphs/Olaqueus_new2.pdf]

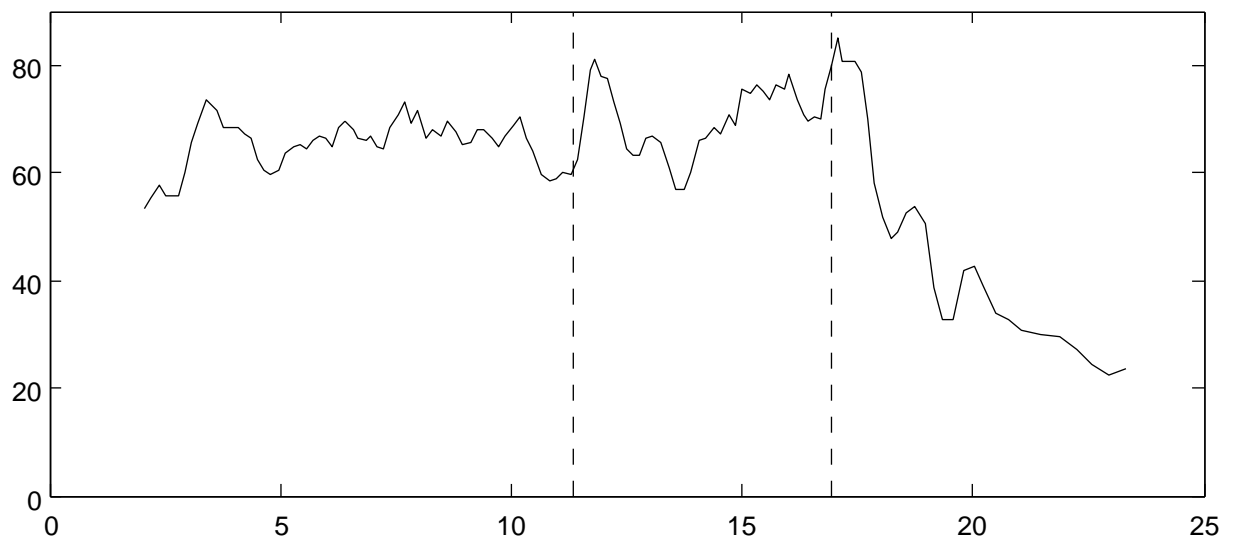

Supplement: Supplementary file 3 [file DataSheet2.ZIP › outputgraphs/Slatimanus_bandspeed.pdf]
